# The L-shaped association between body roundness index and all-cause mortality in osteoporotic patients: a cohort study based on NHANES data

**DOI:** 10.3389/fnut.2025.1538766

**Published:** 2025-01-20

**Authors:** Ziyao Ding, Wenbo Li, Haixu Qi, Tianci Fang, Qirui Zhu, Xinzhe Qu, Changchang Chen, Jun Sun, Yong Pang

**Affiliations:** ^1^First Clinical Medical College, Xuzhou Medical University, Xuzhou, Jiangsu, China; ^2^Key Laboratory of Bone Tissue Regeneration and Digital Medicine, Xuzhou Medical University, Xuzhou, China; ^3^Department of Orthopedics, The Affiliated Hospital of Xuzhou Medical University, Xuzhou, China; ^4^Department of Orthopedics, The First Affiliated Hospital of Soochow University, Suzhou, China

**Keywords:** body roundness index (BRI), bone mineral density (BMD), osteoporosis, NHANES, all-cause mortality

## Abstract

**Purpose:**

This study aims to investigate the relationship between the body roundness index (BRI) and overall mortality rates in individuals with osteoporosis (OP), utilizing information sourced from the NHANES database, in order to assess BRI’s capability as an indicator for predicting mortality risk.

**Methods:**

Data from NHANES (2005 to 2010, 2013–2014, and 2017–2018) were analyzed, including 1,596 osteoporotic individuals aged 50 and above. BRI was calculated based on waist circumference (WC) and height, categorizing participants into high (>4.07) and low (≤4.07) BRI groups. To analyze the relationship between BRI and mortality while accounting for important covariates, we employed weighted Cox proportional hazards models, conducted Kaplan–Meier survival analyses, and utilized restricted cubic splines (RCS).

**Results:**

Higher BRI was significantly associated with better long-term survival, showing an “L”-shaped nonlinear inverse relationship with mortality, with a threshold at BRI = 5. In subgroup analyses, this association remained relatively stable.

**Conclusion:**

The “L”-shaped association between BRI and mortality indicates that BRI may serve as a useful indicator for evaluating mortality risk in patients with OP, thereby informing clinical interventions and public health approaches.

## 1 Introduction

In 1994, the World Health Organization described osteoporosis (OP) as a systemic skeletal disease characterized by structural decay and reduced bone density, significantly heightening the risk of fractures, disability, and premature mortality if left unmanaged (1, 2). This condition not only imposes a growing burden on global healthcare systems and socio-economic resources, especially with aging populations, but also presents unique challenges in predicting mortality due to its multifaceted impact on health and quality of life (3). Unlike many chronic conditions, the interplay between frailty, fracture susceptibility, and systemic comorbidities in OP amplifies the complexity of assessing survival outcomes, necessitating a more nuanced approach to risk stratification (4).

While body mass index (BMI) is a widely recognized measure for evaluating obesity, it fails to accurately represent body fat content or its distribution (5, 6). This limitation is particularly pronounced in clinical scenarios like OP, where body composition plays a critical role in bone health and fracture risk. Recent studies have highlighted fat distribution, particularly the ratio of visceral to subcutaneous fat, as a superior predictor of mortality risks compared to BMI alone (7). To improve abdominal obesity assessment, Thomas et al. (8) introduced a novel measure known as the body roundness index (BRI), based on an elliptical model to measure waist circumference (WC) more accurately, thus providing a better estimation of visceral fat levels and overall body fat percentage. The BRI offers a more accurate estimation of visceral fat levels and overall body fat percentage, demonstrating superior predictive capabilities for various clinical outcomes, including cardiometabolic disorders, renal failure, and cancer (9–11). Longitudinal studies have consistently underscored the link between elevated BRI values and increased mortality, further establishing its relevance in long-term health monitoring (12).

Despite extensive research on the relationships between obesity indices such as BMI and bone health—particularly bone mineral density (BMD) and OP (13)—there remains a significant knowledge gap in understanding how body shape metrics, like the BRI, influence long-term survival in osteoporotic populations. This gap is particularly critical given the distinct interplay of body composition, metabolic health, and fracture-related complications in OP. To address this, our study will leverage the NHANES database to explore the association between the BRI and all-cause mortality in individuals with OP, providing novel insights into mortality risk prediction for this vulnerable population.

## 2 Materials and methods

### 2.1 Data source

This research utilizes information sourced from the NHANES dataset (14). The NHANES survey provides a comprehensive view of nutritional conditions and the health of the U.S. civilian population that is not institutionalized (15). All participants consented before their inclusion in the survey, and the NHANES database is carefully anonymized to ensure that it contains no identifiable information from any participant.

### 2.2 Mortality recognition and follow-up

The vital status of each participant, whether alive or deceased, was ascertained through the linkage of NHANES data with the National Death Index (NDI). We determined the duration of follow-up for each participant, extending from the date of their NHANES examination until either 31 December 2019 or the date of their demise, whichever occurred first, as reported in Yang et al. (16). The classification of mortality causes adhered to the guidelines established by the International Classification of Diseases, 10th Revision (ICD-10), which provides a standardized framework for recording and coding various health conditions and causes of death.

### 2.3 Research population

**Figure 1:**
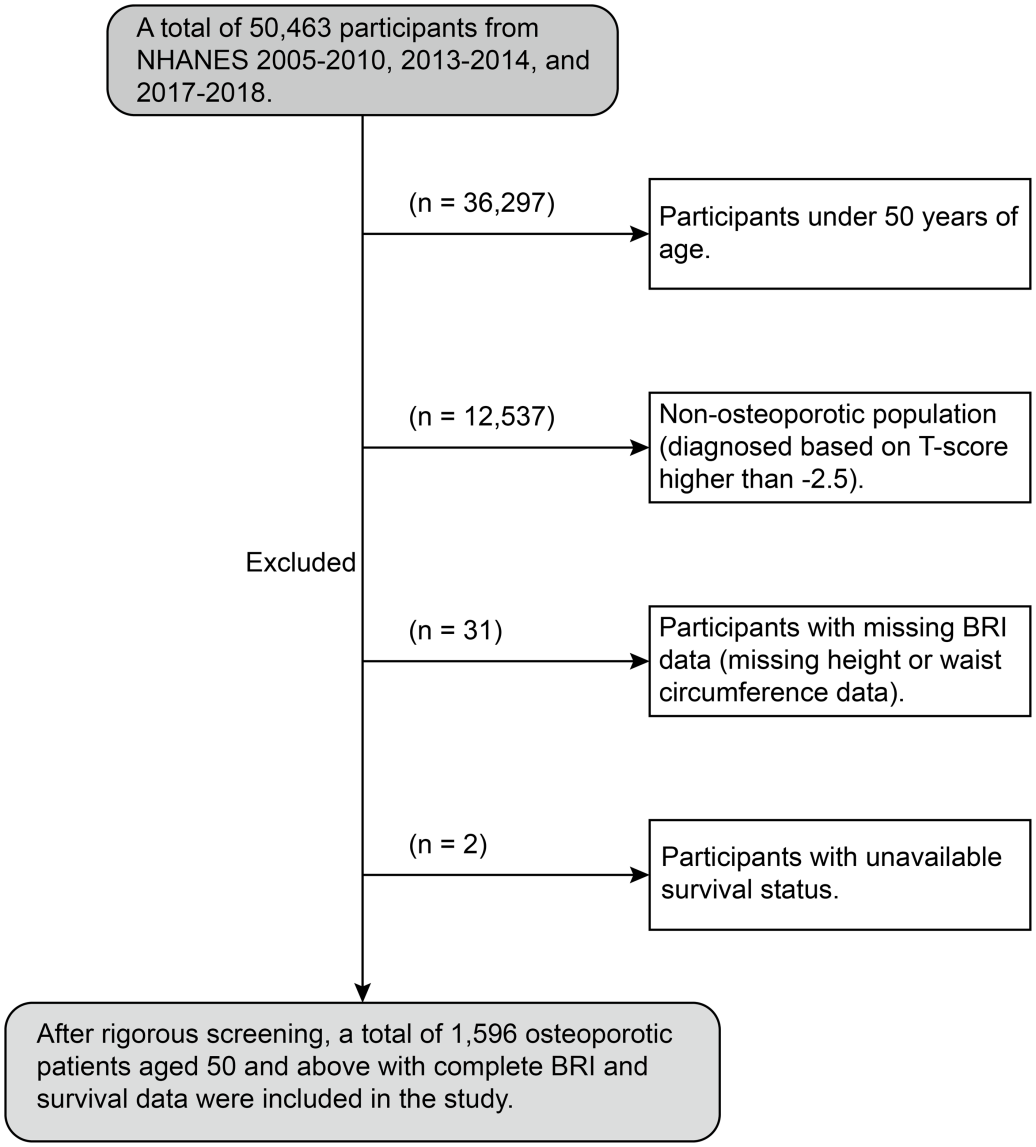
The flow chart of participants in the current study.

The research incorporates survey data from 2005 to 2010, 2013–2014, and 2017–2018, selected for their detailed bone density measurements in individuals 50 years and older. Out of an initial pool of 50,463 participants, the data encompasses interviews, blood analysis, and lab results. Exclusions were made for individuals younger than 50 (*N* = 36,297), those without OP (*N* = 12,573), those lacking BRI data (*N* = 31), or those whose survival status was unknown (*N* = 2). After these criteria were applied, the final sample size for the study was narrowed down to 1,596 participants (as shown in Figure 1).

### 2.4 Definition of BMD measurement and OP

BMD assessments within the NHANES program were conducted at mobile examination centers (MECs) utilizing state-of-the-art dual-energy X-ray absorptiometry (DXA) technology. The measurements were conducted using an instrument, the Hologic Discovery Model A densitometer, which is widely recognized for its exceptional precision and reliability. To ensure precision and uniformity, all procedures were conducted by certified radiographers who had undergone specialized NHANES training. The primary focus of these assessments was on two crucial skeletal areas: the femoral neck and the lumbar spine (L1-L4). The BMD of the lumbar spine was assessed by averaging the values from vertebrae L1 to L4. These measurements were subsequently converted to *T*-scores, which were derived by subtracting the mean BMD of a young, healthy reference population from the participant’s BMD, and then dividing the result by the standard deviation of BMD within the reference group. The reference group consisted of non-Hispanic White individuals, both male and female, within the age range of 20 to 29 years, with data collected between 2005 and 2008. Sex-specific means and standard deviations were utilized for analysis (17). A diagnosis of OP was made when the *T*-score at either the femoral neck or lumbar spine was ≤−2.5, adhering to established diagnostic criteria and reference standards (18, 19). Additional insights into these diagnostic thresholds and their derivation can be accessed in Supplementary Table S1.

### 2.5 Definition of BRI

The BRI = 364.2–365.5 × √[1 − (WC^2^/(4*π*^2^))/(0.5 × height)^2^], where WC represents waist circumference (8). Both WC and height measurements were meticulously obtained following standardized protocols to guarantee the consistency and precision of the collected data.

### 2.6 Selecting covariates

The selection of covariates is based on clinical judgment and previous research identifying important confounding factors (20, 21). Clinical and demographic characteristics of the participants included in the analysis encompass age group, sex, race/ethnicity (grouped into categories of Mexican American, non-Hispanic White, non-Hispanic Black, and other ethnicities), educational background (categorized as college or higher, high school or equivalent, less than high school), and family PIR (poverty-income ratio, which provides an economic indicator of family income relative to the poverty threshold). In addition, clinical measures such as blood calcium and phosphorus concentrations, vitamin D levels, and lifestyle factors such as height, alcohol intake, BMI, waist measurement, and tobacco use are included. Health conditions such as hypertension, hyperlipidemia, cardiovascular disease (CVD), diabetes, liver disease, malignancy, thyroid dysfunction, and renal insufficiency are also assessed. Participants are identified as smokers if they have ever smoked at least 100 cigarettes. Heavy drinking is defined as consuming at least four alcoholic drinks per day for women or five for men, almost every day, at any point in their lives. Hypertension is diagnosed if the systolic blood pressure is 140 mmHg or higher, if the diastolic blood pressure is 90 mmHg or above, if a doctor has previously diagnosed hypertension, or if antihypertensive medication is currently being used (22). Hyperlipidemia is characterized by a total serum cholesterol level of at least 6.2 mmol/L, a self-reported diagnosis of dyslipidemia confirmed by a healthcare provider, or a medical recommendation to initiate lipid-lowering treatment (23). Diabetes is identified through a confirmed diagnosis by a physician, current treatment with oral hypoglycaemic medications or insulin, an HbA1c level of at least 6.5%, or A fasting plasma glucose level of 7.0 mmol/L or above (24). CVD encompasses conditions including stroke, myocardial infarction, congestive heart failure, coronary heart disease, and angina pectoris, as reported in participant questionnaires. Data on kidney disease, malignancies, liver disease, and thyroid disease are also collected through self-report surveys.

### 2.7 Statistical analysis

This study uses the NHANES complex sampling framework, which incorporates stratification, clustering, and appropriate sample weights (calculated as the full-sample 2-year MEC examination weight divided by 5). For baseline group comparisons, categorical variables were analyzed using survey-weighted chi-squared tests, while continuous variables were assessed using survey-weighted linear regression models. The optimal cut-off for the BRI was determined using the “maxstat” R package (25), which allowed participants to be divided into low and high BRI groups. Survival rates in the osteoporotic group were estimated using Kaplan–Meier analysis, and statistical significance was evaluated through the log-rank test. To investigate the independent relationship between BRI and all-cause mortality in patients with osteoporosis, researchers applied survey-weighted Cox proportional hazards regression models. Three models were evaluated: Model 1 (univariate regression model), Model 2 (adjusted for age group, gender, and race), and Model 3 (adjusted for race, hypertension, education level, gender, calcium, 25-OHD, smoking status, diabetes, cancer, drinking status, age group, liver disease, hyperlipidemia, kidney failure, and CVD). Non-linear associations were investigated using survey-weighted restricted cubic splines (RCS), with the final model chosen by identifying the one with the minimum Akaike information criterion (AIC) value, typically utilizing three nodes. The inflection point was identified from the spline curve, and a segmented Cox regression model was fitted around this point. The presence of the inflection point was validated by comparing the segmented model to the unsegmented version using a log-likelihood ratio test (25). Stratified and interaction analyses were performed to examine the effect of demographic characteristics, alcohol consumption, smoking habits, and comorbid conditions on the association between risk of death and BRI. Sensitivity analyses were carried out on the data set with cases of death within the first 2 years removed and on the non-imputed data set to verify the stability of the conclusions. Missing data, which accounted for less than 30% of the dataset and were presumed to be missing at random, were addressed through imputation using the k-nearest neighbors (kNN) method implemented via the VIM package in R (26). All statistical analyses were conducted utilizing R software (version 4.3.1) and EmpowerStats, with a two-sided *p*-value threshold of less than 0.05 considered indicative of statistical significance.

## 3 Results

### 3.1 Baseline characteristics of study participants

**Figure 2:**
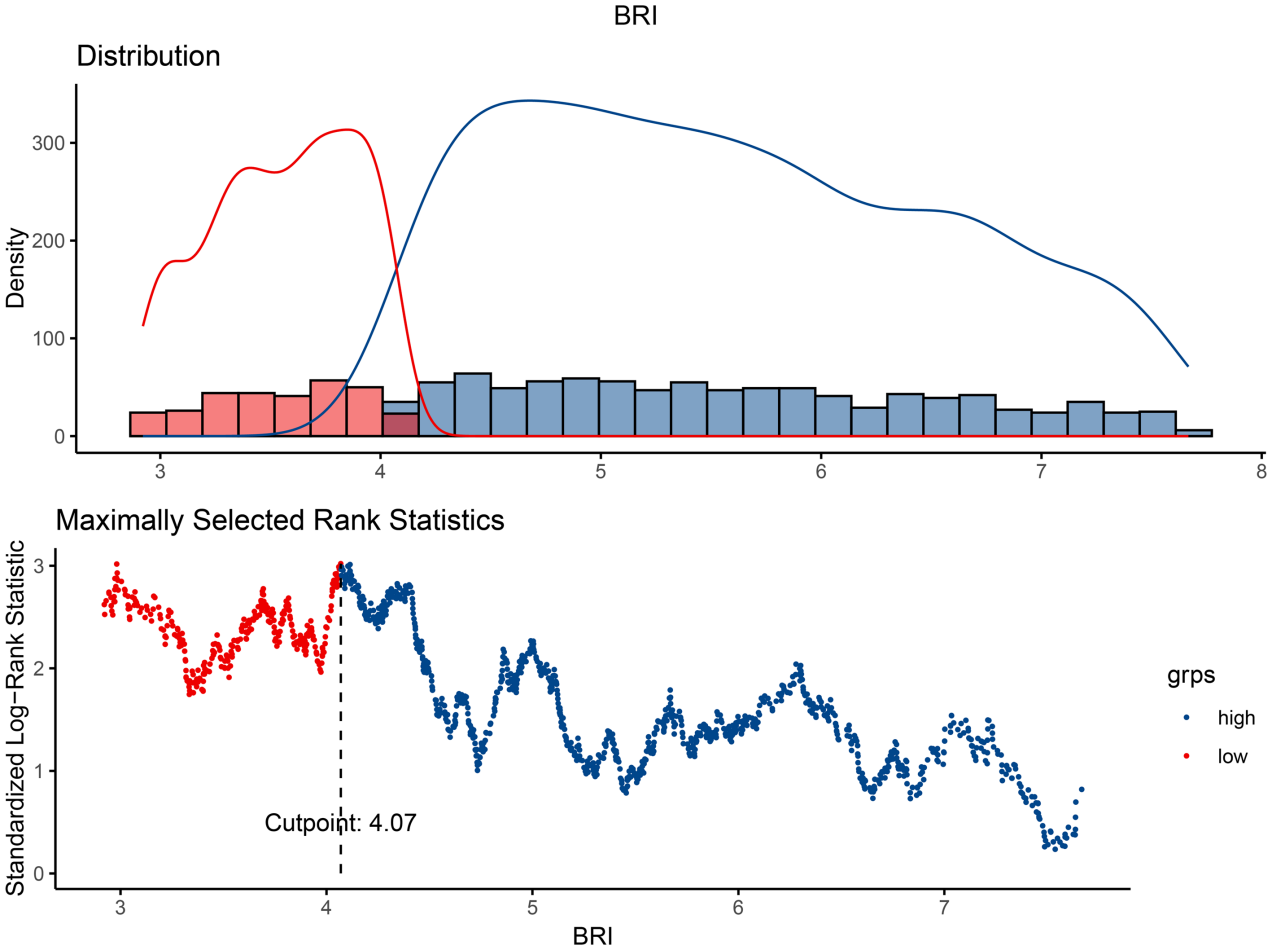
The cutoff point was calculated using the maximally selected rank statistics based on the “maxstat” package.

This study examined data from 1,596 individuals aged 50 and above diagnosed with OP. The optimal cut-off value for the BRI about survival outcomes was identified as 4.07 using the “maxstat” R package. Participants were then categorized into two groups based on this threshold: a high BRI group (BRI >4.07, *n* = 1,126) and a low BRI group (BRI ≤4.07, *n* = 470), as illustrated in Figure 2. There were clear differences between the two groups. Those in the high BRI category were generally older, and more Mexican-Americans had lower levels of formal education, lower household income, shorter stature, larger WC, and higher BMI. They also had lower serum calcium concentrations, higher BMD *T*-scores, and an increased occurrence of conditions such as diabetes, cardiovascular disease, hyperlipidemia, and hypertension. Despite these characteristics, the high BRI group exhibited reduced mortality rates. Further details are shown in Table 1.

**Table 1:** Baseline characteristics of included participants (weighted).

Characteristics	Total	Low BRI	High BRI	*p*-value
Unweighted number	(*n* = 1,596)	(*n* = 470)	(*n* = 1,126)	
Weighted number	*N* = 10,830,756	*N* = 3,739,778	*N* = 7,090,978	
Age group, %				0.0110
50–79	79.39 (76.90, 81.67)	83.45 (79.42, 86.83)	77.24 (74.11, 80.10)	
80+	20.61 (18.33, 23.10)	16.55 (13.17, 20.58)	22.76 (19.90, 25.89)	
Gender, %				0.3074
Male	27.41 (24.30, 30.76)	29.44 (24.64, 34.75)	26.34 (22.63, 30.41)	
Female	72.59 (69.24, 75.70)	70.56 (65.25, 75.36)	73.66 (69.59, 77.37)	
Race, %				<0.0001
Mexican American	5.22 (3.97, 6.84)	1.79 (1.12, 2.85)	7.03 (5.35, 9.17)	
Non-Hispanic White	78.33 (75.03, 81.31)	80.93 (76.22, 84.89)	76.96 (73.16, 80.37)	
Non-Hispanic Black	3.99 (3.12, 5.10)	4.15 (2.98, 5.76)	3.91 (2.90, 5.26)	
Other race	12.45 (10.44, 14.80)	13.13 (10.02, 17.02)	12.10 (9.84, 14.79)	
Education level, %				0.0275
Less than high school	23.28 (20.59, 26.21)	17.67 (13.35, 23.02)	26.24 (22.98, 29.79)	
High school or equivalent	26.50 (23.21, 30.06)	29.13 (23.35, 35.67)	25.11 (22.00, 28.49)	
College or above	50.22 (46.51, 53.93)	53.19 (45.64, 60.61)	48.65 (44.29, 53.03)	
Family PIR	2.79 (2.66, 2.92)	2.94 (2.76, 3.12)	2.71 (2.57, 2.86)	0.0269
Height, cm	161.20 (160.57, 161.83)	163.18 (162.23, 164.13)	160.16 (159.36, 160.96)	<0.0001
WC, cm	92.54 (91.48, 93.60)	79.65 (78.82, 80.48)	99.34 (98.40, 100.28)	<0.0001
BMI, kg/cm^2^	25.42 (25.07, 25.78)	20.96 (20.70, 21.23)	27.77 (27.39, 28.16)	<0.0001
Calcium, mg/dL	9.43 (9.40, 9.46)	9.47 (9.43, 9.52)	9.41 (9.38, 9.45)	0.0165
Phosphorus, mg/dL	3.82 (3.78, 3.86)	3.85 (3.78, 3.91)	3.81 (3.76, 3.86)	0.3970
25-OHD, nmol/L	75.61 (72.93, 78.28)	77.22 (73.05, 81.38)	74.76 (71.87, 77.65)	0.2746
Lumber *T*-score	−2.32 (−2.39, −2.24)	−2.43 (−2.56, −2.30)	−2.25 (−2.34, −2.17)	0.0290
Femoral *T*-score	−2.65 (−2.69, −2.60)	−2.74 (−2.80, −2.68)	−2.60 (−2.65, −2.54)	0.0002
Smoker, %				0.0567
No	53.31 (49.71, 56.88)	48.18 (41.48, 54.95)	56.01 (51.71, 60.23)	
Yes	46.69 (43.12, 50.29)	51.82 (45.05, 58.52)	43.99 (39.77, 48.29)	
Heavy drinker, %				0.6605
No	88.41 (86.10, 90.38)	87.64 (82.53, 91.41)	88.82 (85.92, 91.18)	
Yes	11.59 (9.62, 13.90)	12.36 (8.59, 17.47)	11.18 (8.82, 14.08)	
Hypertension, %				0.0021
No	41.80 (38.23, 45.46)	48.80 (42.99, 54.64)	38.11 (34.05, 42.35)	
Yes	58.20 (54.54, 61.77)	51.20 (45.36, 57.01)	61.89 (57.65, 65.95)	
Dyslipidemia, %				<0.0001
No	44.82 (41.54, 48.14)	58.05 (51.24, 64.56)	37.84 (34.24, 41.58)	
Yes	55.18 (51.86, 58.46)	41.95 (35.44, 48.76)	62.16 (58.42, 65.76)	
CVD, %				0.0016
No	81.66 (78.92, 84.12)	86.51 (82.29, 89.85)	79.10 (75.90, 81.99)	
Yes	18.34 (15.88, 21.08)	13.49 (10.15, 17.71)	20.90 (18.01, 24.10)	
Diabetes, %				<0.0001
No	84.75 (82.25, 86.96)	92.33 (88.73, 94.84)	80.76 (77.76, 83.43)	
Yes	15.25 (13.04, 17.75)	7.67 (5.16, 11.27)	19.24 (16.57, 22.24)	
Thyroid disease, %				0.2002
No	78.12 (75.04, 80.91)	81.05 (75.62, 85.50)	76.57 (72.26, 80.39)	
Yes	21.88 (19.09, 24.96)	18.95 (14.50, 24.38)	23.43 (19.61, 27.74)	
Liver disease, %				0.9813
No	93.90 (91.43, 95.69)	93.87 (90.25, 96.20)	93.92 (90.57, 96.13)	
Yes	6.10 (4.31, 8.57)	6.13 (3.80, 9.75)	6.08 (3.87, 9.43)	
Cancer, %				0.2036
No	78.54 (75.74, 81.10)	76.32 (71.45, 80.59)	79.71 (76.39, 82.67)	
Yes	21.46 (18.90, 24.26)	23.68 (19.41, 28.55)	20.29 (17.33, 23.61)	
Kidney failure, %				0.4485
No	93.67 (91.22, 95.48)	94.53 (90.10, 97.05)	93.22 (90.79, 95.04)	
Yes	6.33 (4.52, 8.78)	5.47 (2.95, 9.90)	6.78 (4.96, 9.21)	
Vital status, %				0.0104
Alive	72.93 (70.02, 75.66)	68.72 (64.16, 72.93)	75.15 (71.76, 78.27)	
Deceased	27.07 (24.34, 29.98)	31.28 (27.07, 35.84)	24.85 (21.73, 28.24)	

Family PIR, family poverty income ratio; WC, waist circumference; BMI, body mass index; CVD: cardiovascular disease. For continuous variables: survey-weighted mean (95% CI), *p*-value was by survey-weighted linear regression, for categorical variables: survey-weighted percentage (95% CI), *p*-value was by survey-weighted chi-square test.

### 3.2 Kaplan–Meier survival analysis

**Figure 3:**
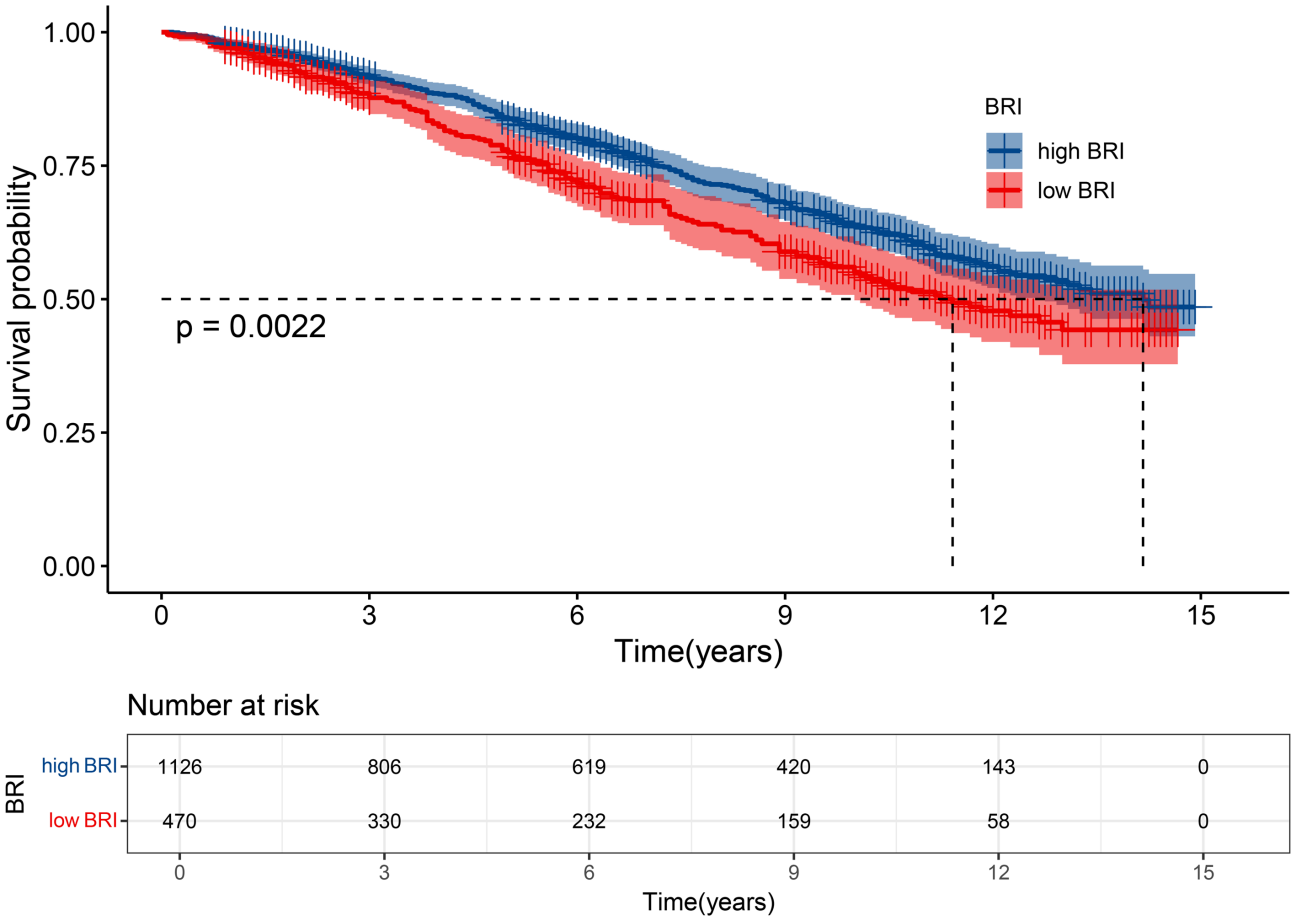
Kaplan–Meier curves of the survival rate with high (>4.07) and low (≤4.07) BRI values.

Analysis was used to assess the effect of BRI on survival outcomes in the osteoporotic population. The results showed that participants with a higher BRI had significantly improved survival compared to those with a lower BRI, with statistical significance achieved (*p* = 0.0022). In addition, individuals in the low BRI group had a markedly shorter median survival time compared to those in the high BRI group. These survival patterns are illustrated in Figure 3.

### 3.3 Association between BRI and all-cause mortality in the osteoporotic population

The study tracked a cohort of 1,596 individuals diagnosed with OP over a median follow-up duration of 6.25 years (interquartile range: 2.67–10.17 years) to investigate the link between all-cause mortality and the BRI. Throughout the observation period, 496 participants (31.1%) passed away. To rigorously analyze this association, researchers applied a weighted Cox proportional hazards regression model, with comprehensive findings presented in Table 2. This methodological approach highlights the nuanced dynamics between body composition and mortality outcomes in osteoporotic populations. In the unadjusted analysis (Model 1), a higher BRI demonstrated a non-significant association with reduced mortality risk, yielding an HR of 0.97 (95% CI: 0.92–1.03, *p* = 0.3934). When adjusted for demographic variables (Model 2), each unit increase in BRI corresponded to a 5% lower risk of all-cause mortality (HR: 0.95, 95% CI: 0.89–1.00, *p* = 0.0630); however, this outcome failed to achieve statistical significance, suggesting that the observed trend may be due to chance rather than a definitive association, warranting further investigation to clarify its implications. In the fully adjusted analysis (Model 3), which incorporated comorbidities, a one-unit rise in BRI was significantly linked to an 11% reduction in mortality risk (HR: 0.89, 95% CI: 0.83–0.95, *p* = 0.0001). Analysis stratified by BRI categories revealed that individuals in the high BRI group consistently showed a markedly lower risk of all-cause mortality among individuals with higher BRI levels when compared to those in the low BRI group. This inverse relationship was observed across all models: Model 1 (HR: 0.81, 95% CI: 0.65–1.00, *p* = 0.0459), Model 2 (HR: 0.67, 95% CI: 0.56–0.81, *p* < 0.0001), and Model 3 (HR: 0.57, 95% CI: 0.47–0.71, *p* < 0.0001). These findings underscore a robust and independent negative association between higher BRI and decreased all-cause mortality risk among osteoporotic patients in the study population.

**Table 2:** Association between BRI and all-cause mortality in osteoporotic adults (weighted).

Characteristic	Model 1		Model 2		Model 3	
	HR (95% CI)	*p*-value	HR (95% CI)	*p*-value	HR (95% CI)	*p*-value
All-cause mortality						
BRI (continuous)	0.97 (0.92, 1.03)	0.3934	0.95 (0.89, 1.00)	0.0630	0.89 (0.83, 0.95)	0.0001
BRI (category)						
Low	Ref.		Ref.		Ref.	
High	0.81 (0.65, 1.00)	0.0459	0.67 (0.56, 0.81)	<0.0001	0.57 (0.47, 0.71)	<0.0001

Model 1, unadjusted; Model 2, adjusted for age group, gender, race; Model 3, adjusted for age group, gender, race, education level, family PIR, smoker, heavy drinker, calcium, 25-OHD, hypertension, dyslipidemia, cardiovascular disease, diabetes, liver disease, cancer, and kidney failure.

### 3.4 Weighted constrained cubic spline and threshold impact model

**Figure 4:**
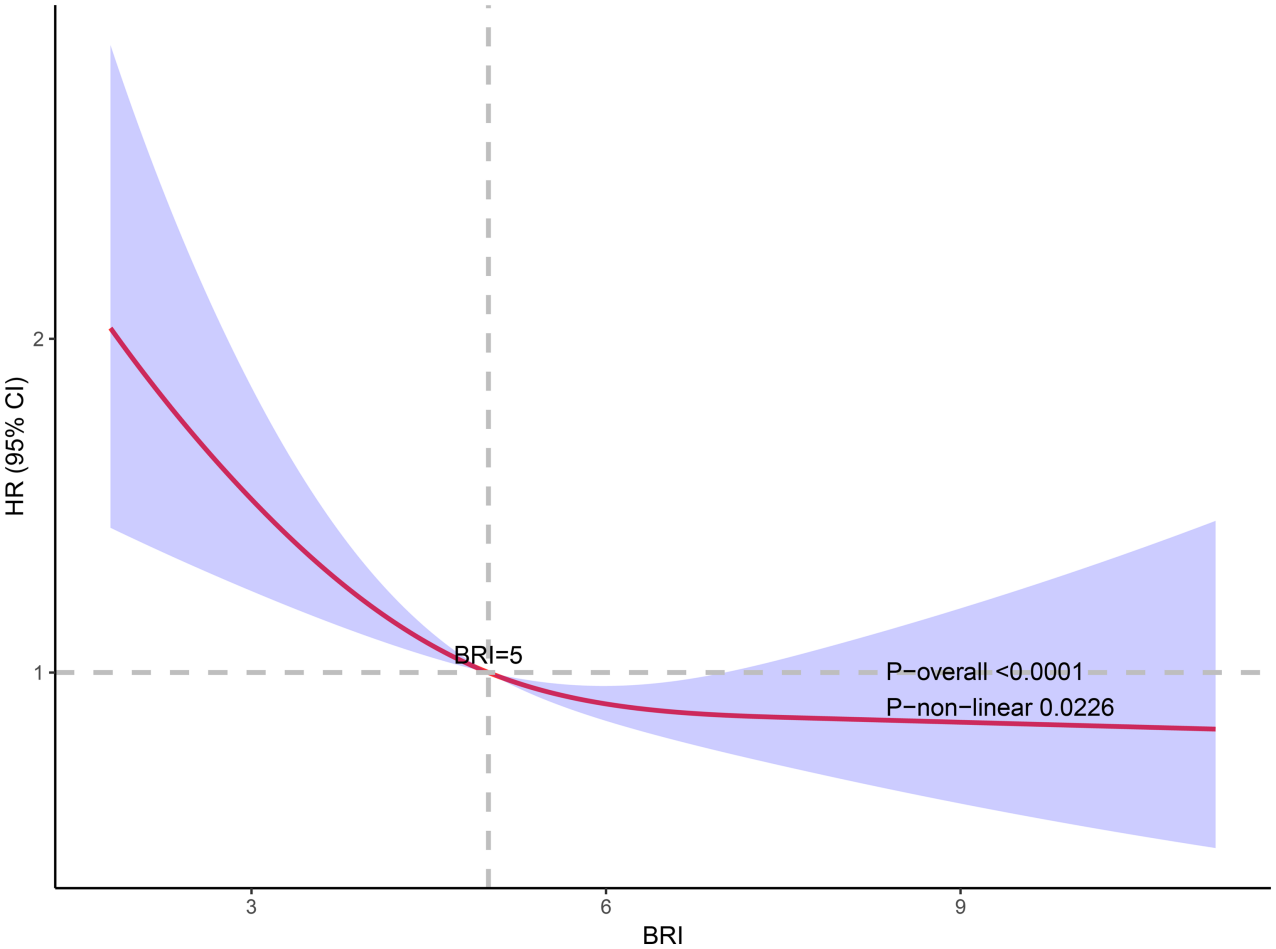
The association of BRI with all-cause mortality. Adjusted for age group, gender, race, family PIR, education level, calcium, 25-OHD, smoking status, drinking status, hypertension, hyperlipidemia, CVD, diabetes, liver disease, cancer, and kidney failure.

Utilizing weighted restricted cubic spline analysis alongside segmented Cox regression models, this study explored the potential nonlinear relationship between BRI and all-cause mortality in individuals diagnosed with OP. This innovative approach allowed for a detailed examination of threshold effects and dynamic patterns within the data, offering new insights into the interplay between body composition and mortality risk. After accounting for all covariates, the RCS analysis identified a significant nonlinear association, characterized by an “L”-shaped curve, between BRI and mortality risk (*p* for non-linearity = 0.0226). This relationship is visually depicted in Figure 4. Further analysis of the curve pinpointed a BRI value of 5 as the critical inflection point, marking a shift in the pattern of the association. To explore the threshold effect, segmented Cox proportional hazards regression was conducted separately for BRI values below and above the identified inflection point. The analysis revealed a significant log-likelihood ratio test result (*p* = 0.0030), providing strong evidence of a threshold in the relationship between BRI and mortality. For BRI values below 5 (BRI <5), an elevated BRI was strongly linked to reduced mortality risk, with a hazard ratio of 0.79 [95% confidence interval (CI): 0.70–0.88, *p* < 0.0001]. However, for BRI values of 5 or greater (BRI ≥5), the inverse relationship was no longer significant, with an HR of 0.97 (95% CI: 0.87–1.09, *p* = 0.6198). These findings confirm that the protective effect of BRI on mortality diminishes beyond the inflection point. These results suggest the existence of an optimal BRI range, below which higher BRI values are associated with improved survival outcomes in individuals with OP, while values above the threshold do not confer any additional benefit. Full details are shown in Table 3.

**Table 3:** Threshold effect analysis of BRI and all-cause mortality in osteoporotic adults (weighted).

	HR (95% CI)	*p*-value
All-cause mortality		
Model I	0.89 (0.83, 0.95)	0.0011
Model II		
Inflection point (K)	5.00	
<K point effect 1	0.79 (0.70, 0.88)	<0.0001
>K point effect 2	0.97 (0.87, 1.09)	0.6198
*p* for log-likelihood ratio		0.0030

Model I: non-segmented regression model; Model II: segmented regression model. Adjusted for age group, gender, race, education level, family PIR, smoker, heavy drinker, calcium, 25-OHD, hypertension, dyslipidemia, cardiovascular disease, diabetes, liver disease, cancer, and kidney failure.

### 3.5 Analysis of subgroup

**Figure 5:**
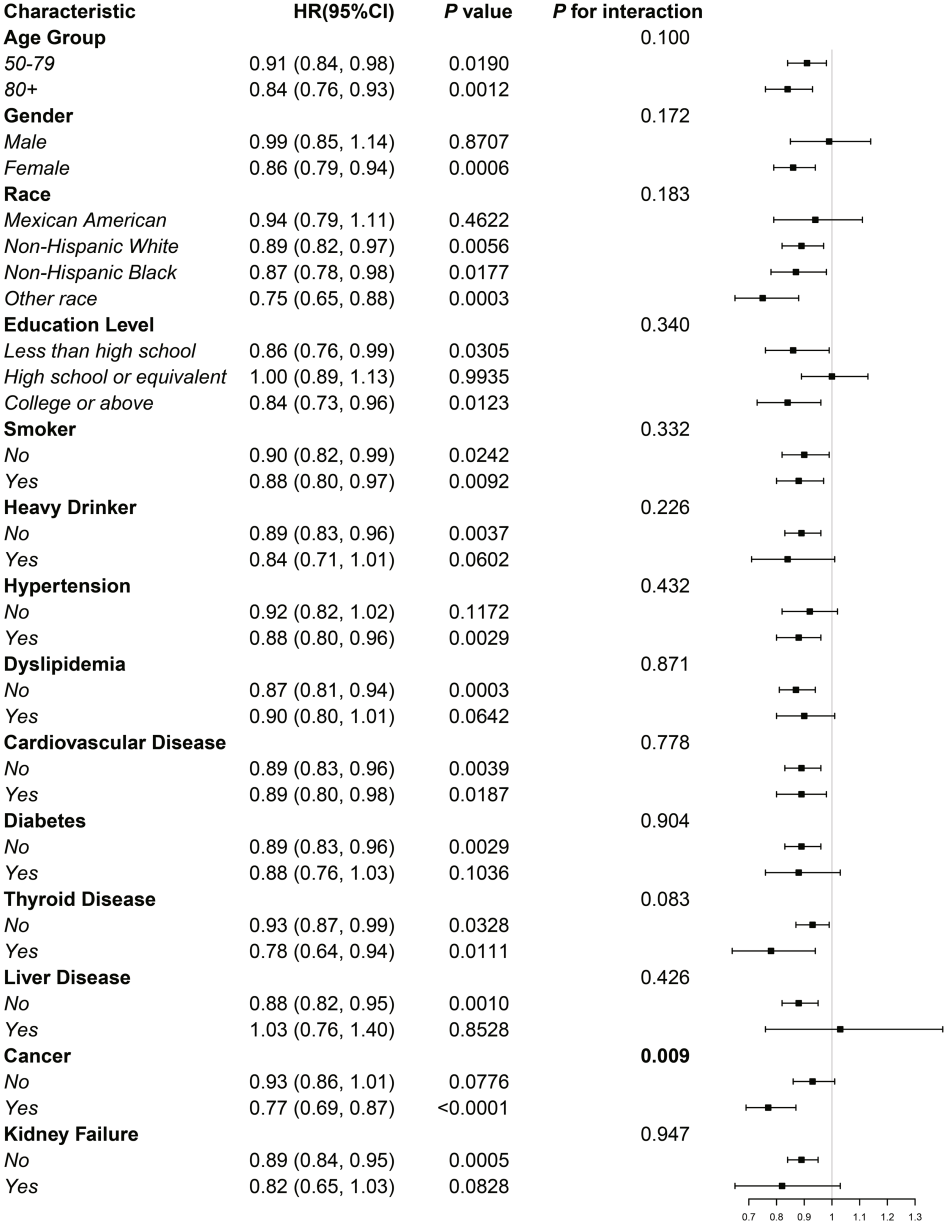
Subgroup analysis of the association between BRI and all-cause mortality in the osteoporotic population.

To assess variations in the relationship between BRI and all-cause mortality across different population groups, researchers conducted interaction tests and subgroup analyses. Cox proportional hazard models with weighting were applied, with adjustments made for all covariates except the specific variable used to define each subgroup. The findings revealed a significant interaction effect between BRI and all-cause mortality within the cancer subgroup (*p* for interaction = 0.009). However, no significant interaction effects were detected in the remaining subgroups. A visual representation of these outcomes is provided in Figure 5. A more in-depth analysis was conducted to examine the cancer subgroup in greater detail. The findings revealed a strong and statistically significant negative correlation between BRI and all-cause mortality among individuals with cancer. Specifically, for every one-unit increase in BRI, a 23% decrease in mortality risk was observed (HR was 0.77, with a 95% CI of 0.69 to 0.87, and a *p*-value of less than 0.0001.). These findings underscore the strong protective association between higher BRI levels and reduced mortality within this subgroup. These results highlight that the independent inverse association between BRI and all-cause mortality, which is broadly consistent across different subgroups of the osteoporotic population, is particularly pronounced in patients with cancer. This suggests a potential subgroup-specific effect that warrants further investigation.

### 3.6 Analysis of sensitivity

To strengthen the robustness of our findings, a sensitivity analysis was conducted by excluding individuals who died during the first 2 years of follow-up. This analysis further confirmed the inverse relationship between BRI and all-cause mortality. In the fully adjusted Model 3, elevated BRI values consistently exhibited a significant association with reduced mortality risk, with each unit increase in BRI corresponding to a 9% decrease in the likelihood of death (HR: 0.91, 95% CI: 0.84–0.98, *p* = 0.0146). Detailed results are presented in Supplementary Table S2. Further stratified regression analysis based on BRI categories revealed a marked reduction in mortality risk for individuals in the high BRI group. In the fully adjusted model, participants in this category demonstrated an HR of 0.59 (95% CI: 0.46–0.74, *p* < 0.0001), indicating a substantially lower risk of all-cause mortality compared to those in the low BRI group. Additionally, an analysis of the non-imputed dataset, detailed in Supplementary Table S3, yielded consistent results. After full adjustment for confounding factors, BRI remained inversely associated with all-cause mortality. These findings underscore the strength of the inverse relationship between BRI and all-cause mortality in individuals with osteoporosis. The consistency of this association across multiple analytical approaches reinforces the hypothesis that elevated BRI values may confer a survival advantage within this patient population.

## 4 Discussion

Various analytical approaches were employed in the study to investigate the relationship between BRI and all-cause mortality in older adults with OP. Utilizing data from 1,596 individuals with OP in the NHANES database, the findings indicated that participants with higher BRI values experienced improved long-term survival compared to those with lower BRI scores. Regression models demonstrated a significant inverse relationship between BRI and mortality risk, independent of other covariates. Additional analyses, including RCS and threshold effect evaluations, identified an L-shaped nonlinear pattern, highlighting a threshold effect. Specifically, increases in BRI below a critical point were linked to reduced mortality risk, whereas further increases beyond this threshold had little impact on survival outcomes.

Patients with OP generally face reduced survival due to compromised bone integrity and systemic metabolic imbalances. Fractures, particularly of the hip and spine, significantly increase the associated complications and the risk of mortality (27). In addition, malnutrition and co-morbidities such as cardiovascular disease and diabetes exacerbate the decline in survival (28). Adherence to treatment regimens and lifestyle changes is also a challenge for many patients (29). Previous studies, such as those by Cai et al. (30) and Shangguan et al. (31), have linked malnutrition and lower BMD to higher mortality in osteoporotic individuals using NHANES data.

Abdominal obesity, though recognized as a risk factor for mortality and cardiovascular events (32, 33), remains a significant concern, the BRI—derived from the eccentricity of an elliptical body model using height and WC—provides a nuanced measure of abdominal adiposity (8). Previous studies have found U-shaped associations between BRI and mortality in general populations. For instance, a similar relationship between BRI and both all-cause and cardiovascular mortality was observed by Wang et al. (34) through their analysis of NHANES data, and Zhou et al. (35) observed a similar relationship, with increased mortality risk at both low and high BRI levels. However, these analyses did not focus on individuals with OP, a gap that this study addresses.

Our findings revealed a distinct L-shaped non-linear association between the body roundness index (BRI) and all-cause mortality in osteoporotic patients. Specifically, when BRI values were below 5, a strong inverse relationship was observed, suggesting that lower BRI levels may be indicative of malnutrition, muscle wasting, and diminished physical resilience, all of which contribute to increased mortality risk. This aligns with previous research, such as that of Shangguan et al. (31), which highlights the significant role of malnutrition in elevating mortality rates among osteoporotic populations. Improved nutritional status and greater muscle mass have been shown to reduce fracture-related mortality, further supporting the importance of maintaining adequate body composition (21). In contrast, as BRI values exceeded 5, the inverse relationship attenuated, and the mortality risk plateaued, pointing to a potential “threshold effect” in this association.

The observed non-linear relationship can be attributed to the interplay of mechanical, nutritional, and molecular mechanisms. Mechanically, higher body weight exerts greater stress on bones, stimulating adaptive remodeling according to Wolff’s law, thereby enhancing bone density and strength (36, 37). Concurrently, increased weight is often accompanied by greater muscle mass, which generates tensile forces on the bone that promote osteogenesis, improving skeletal stability and reducing fracture risks (38, 39). Nutritionally, visceral fat stores provide critical energy reserves during periods of illness or fracture recovery (40), while also serving as a repository for fat-soluble vitamins, including vitamin D. Sufficient levels of vitamin D play a crucial role in calcium metabolism, supporting bone mineralization and strength (41). At the molecular level, visceral fat acts as a vital endocrine organ, secreting adipokines such as leptin and adiponectin. Leptin regulates bone metabolism via the hypothalamic-pituitary-gonadal axis, while adiponectin exerts anti-inflammatory effects, mitigating bone resorption (42). Additionally, visceral fat facilitates the peripheral conversion of androgens into estrogens, which inhibit osteoclast activity, thereby reducing bone loss (43).

However, excessive visceral fat accumulation can lead to metabolic imbalances and increased cardiovascular burdens, potentially offsetting its protective effects (44, 45). This dual role of visceral fat may explain the observed plateau in mortality risk at higher BRI levels. Collectively, these findings highlight the intricate balance between mechanical, nutritional, and molecular factors that mediate the relationship between adiposity, bone health, and mortality risk in osteoporotic patients.

The subgroup analyses and interaction tests confirmed the robustness of the inverse relationship between BRI and mortality in different populations, with the strongest association observed in the cancer subgroup. In cancer patients, cachexia and malnutrition may explain the increased survival benefit associated with higher BRI (46). However, to determine whether these associations reflect causality, further research is required.

This study has several strengths. First, it used a variety of analytical techniques and a long follow-up period, which increased the reliability of its findings. Second, the use of sample weights ensured broader applicability to the general population. Moreover, this study is the first to recognize an L-shaped non-linear relationship between BRI and mortality in osteoporotic patients, providing valuable insights for health assessment and management in this group. The extensive adjustment for potential confounders further strengthens the validity of the results.

However, this study is not without its limitations. First, the NHANES dataset lacks detailed diagnostic information on osteoporosis, which restricts the depth of the analysis. Additionally, the dataset does not include quantitative assessments of comorbidities, with some conditions relying on self-reported diagnoses, potentially introducing reporting bias. While this is a cohort study, the retrospective design inherently limits the ability to establish causal relationships, which remains a key challenge in such studies. Furthermore, the relatively small sample size constrained our capacity to investigate cause-specific mortality in greater detail. Finally, although the cohort design provides robust evidence supporting an association between BRI and mortality, further prospective research is needed to confirm causality and elucidate the underlying mechanisms.

## 5 Conclusion

This large cohort study, conducted in the United States, identified an L-shaped non-linear association between the BRI and all-cause mortality in individuals with osteoporosis over a median follow-up period of 6.25 years. The observed relationship demonstrated a threshold effect, suggesting the presence of an optimal body shape range for this patient population. These findings underscore the potential of BRI as a simple, non-invasive screening tool for assessing mortality risk and identifying high-risk individuals within the osteoporotic population. Moreover, the utility of BRI in health assessment and risk stratification highlights its broader applicability in public health initiatives aimed at reducing mortality and improving overall population health. However, it is important to note that these conclusions lack support from prospective studies, necessitating further research to validate and expand upon these findings.

## Data Availability

Publicly available datasets were analyzed in this study. This data can be found at: https://wwwn.cdc.gov/nchs/nhanes/Default.aspx.

## References

[1] Kanis JA Melton LJ 3rd Christiansen C Johnston CC Khaltaev N. The diagnosis of osteoporosis. J Bone Miner Res. (1994) 9:1137–41. doi: 10.1002/jbmr.5650090802, PMID: 7976495

[2] Giangregorio LM Papaioannou A Macintyre NJ Ashe MC Heinonen A Shipp K. Too fit to fracture: exercise recommendations for individuals with osteoporosis or osteoporotic vertebral fracture. Osteoporos Int. (2014) 25:821–35. doi: 10.1007/s00198-013-2523-2, PMID: 24281053 PMC5112023

[3] LeBoff MS Greenspan SL Insogna KL Lewiecki EM Saag KG Singer AJ. The clinician’s guide to prevention and treatment of osteoporosis. Osteoporos Int. (2022) 33:2049–102. doi: 10.1007/s00198-021-05900-y, PMID: 35478046 PMC9546973

[4] Li W Xie S Zhong S Lan L. The synergistic effect of diabetes mellitus and osteoporosis on the all-cause mortality: a cohort study of an American population. Front Endocrinol. (2023) 14:1308574. doi: 10.3389/fendo.2023.1308574, PMID: 38327903 PMC10849060

[5] Flegal KM Kit BK Orpana H Graubard BI. Association of all-cause mortality with overweight and obesity using standard body mass index categories: a systematic review and meta-analysis. JAMA. (2013) 309:71–82. doi: 10.1001/jama.2012.113905, PMID: 23280227 PMC4855514

[6] Khan I Chong M Le A Mohammadi-Shemirani P Morton R Brinza C. Surrogate adiposity markers and mortality. JAMA Netw Open. (2023) 6:e2334836. doi: 10.1001/jamanetworkopen.2023.34836, PMID: 37728925 PMC10512100

[7] Lee SW Son JY Kim JM Hwang SS Han JS Heo NJ. Body fat distribution is more predictive of all-cause mortality than overall adiposity. Diabetes Obes Metab. (2018) 20:141–7. doi: 10.1111/dom.13050, PMID: 28671751

[8] Thomas DM Bredlau C Bosy-Westphal A Mueller M Shen W Gallagher D. Relationships between body roundness with body fat and visceral adipose tissue emerging from a new geometrical model. Obesity. (2013) 21:2264–71. doi: 10.1002/oby.20408, PMID: 23519954 PMC3692604

[9] Wu L Pu H Zhang M Hu H Wan Q. Non-linear relationship between the body roundness index and incident type 2 diabetes in Japan: a secondary retrospective analysis. J Transl Med. (2022) 20:110. doi: 10.1186/s12967-022-03321-x, PMID: 35255926 PMC8900386

[10] Zhang Y Gao W Ren R Liu Y Li B Wang A. Body roundness index is related to the low estimated glomerular filtration rate in Chinese population: a cross-sectional study. Front Endocrinol. (2023) 14:1148662. doi: 10.3389/fendo.2023.1148662, PMID: 37056676 PMC10086436

[11] Gao W Jin L Li D Zhang Y Zhao W Zhao Y. The association between the body roundness index and the risk of colorectal cancer: a cross-sectional study. Lipids Health Dis. (2023) 22:53. doi: 10.1186/s12944-023-01814-2, PMID: 37072848 PMC10111650

[12] Ding J Chen X Shi Z Bai K Shi S. Association of body roundness index and its trajectories with all-cause and cardiovascular mortality among a Chinese middle-aged and older population: a retrospective cohort study. Front Public Health. (2023) 11:1107158. doi: 10.3389/fpubh.2023.1107158, PMID: 37033022 PMC10076882

[13] Kim KJ Son S Kim KJ Kim SG Kim NH. Weight-adjusted waist as an integrated index for fat, muscle and bone health in adults. J Cachexia Sarcopenia Muscle. (2023) 14:2196–203. doi: 10.1002/jcsm.13302, PMID: 37550773 PMC10570086

[14] Zhang Q Xiao S Jiao X Shen Y. The triglyceride-glucose index is a predictor for cardiovascular and all-cause mortality in CVD patients with diabetes or pre-diabetes: evidence from NHANES 2001–2018. Cardiovasc Diabetol. (2023) 22:279. doi: 10.1186/s12933-023-02030-z, PMID: 37848879 PMC10583314

[15] Liu Y Geng T Wan Z Lu Q Zhang X Qiu Z. Associations of serum folate and vitamin B12 levels with cardiovascular disease mortality among patients with type 2 diabetes. JAMA Netw Open. (2022) 5:e2146124. doi: 10.1001/jamanetworkopen.2021.46124, PMID: 35099545 PMC8804919

[16] Yang F Wang M Chen Y Wu J Li Y. Association of cardio-renal biomarkers and mortality in the U.S.: a prospective cohort study. Cardiovasc Diabetol. (2023) 22:265. doi: 10.1186/s12933-023-01986-2, PMID: 37775738 PMC10542251

[17] Kanis JA Adachi JD Cooper C Clark P Cummings SR Diaz-Curiel M. Standardising the descriptive epidemiology of osteoporosis: recommendations from the epidemiology and quality of life working group of IOF. Osteoporos Int. (2013) 24:2763–4. doi: 10.1007/s00198-013-2413-7, PMID: 23884436 PMC5096926

[18] Bass MA Sharma A Nahar VK Chelf S Zeller B Pham L. Bone mineral density among men and women aged 35 to 50 years. J Am Osteopath Assoc. (2019) 119:357–63. doi: 10.7556/jaoa.2019.064, PMID: 31135863

[19] Looker AC Wahner HW Dunn WL Calvo MS Harris TB Heyse SP. Updated data on proximal femur bone mineral levels of US adults. Osteoporos Int. (1998) 8:468–90. doi: 10.1007/s001980050093, PMID: 9850356

[20] Zhang X Liang J Luo H Zhang H Xiang J Guo L. The association between body roundness index and osteoporosis in American adults: analysis from NHANES dataset. Front Nutr. (2024) 11:1461540. doi: 10.3389/fnut.2024.1461540, PMID: 39430785 PMC11486732

[21] Zhang X Ma N Lin Q Chen K Zheng F Wu J. Body roundness index and all-cause mortality among US adults. JAMA Netw Open. (2024) 7:e2415051. doi: 10.1001/jamanetworkopen.2024.15051, PMID: 38837158 PMC11154161

[22] Tan L Liu Y Liu J Zhang G Liu Z Shi R. Association between insulin resistance and uncontrolled hypertension and arterial stiffness among US adults: a population-based study. Cardiovasc Diabetol. (2023) 22:311. doi: 10.1186/s12933-023-02038-5, PMID: 37946205 PMC10637002

[23] Li Z Zhu G Chen G Luo M Liu X Chen Z. Distribution of lipid levels and prevalence of hyperlipidemia: data from the NHANES 2007–2018. Lipids Health Dis. (2022) 21:111. doi: 10.1186/s12944-022-01721-y, PMID: 36307819 PMC9615374

[24] Li B Chen L Hu X Tan T Yang J Bao W. Association of serum uric acid with all-cause and cardiovascular mortality in diabetes. Diabetes Care. (2023) 46:425–33. doi: 10.2337/dc22-1339, PMID: 36490263

[25] Dong G Gan M Xu S Xie Y Zhou M Wu L. The neutrophil-lymphocyte ratio as a risk factor for all-cause and cardiovascular mortality among individuals with diabetes: evidence from the NHANES 2003–2016. Cardiovasc Diabetol. (2023) 22:267. doi: 10.1186/s12933-023-01998-y, PMID: 37775767 PMC10541705

[26] He W Chen S Fu X Xu L Xie J Wan J. Development and evaluation of interpretable machine learning regressors for predicting femoral neck bone mineral density in elderly men using NHANES data. Biomol Biomed. (2024). doi: 10.17305/bb.2024.10725, PMID: 38972052 PMC11734819

[27] Barnsley J Buckland G Chan PE Ong A Ramos AS Baxter M. Pathophysiology and treatment of osteoporosis: challenges for clinical practice in older people. Aging Clin Exp Res. (2021) 33:759–73. doi: 10.1007/s40520-021-01817-y, PMID: 33742387 PMC8084810

[28] Ebeling PR Nguyen HH Aleksova J Vincent AJ Wong P Milat F. Secondary osteoporosis. Endocr Rev. (2022) 43:240–313. doi: 10.1210/endrev/bnab028 34476488

[29] Burns RB Rosen H Berry S Smetana GW. How would You manage this patient with osteoporosis?: grand rounds discussion from Beth Israel Deaconess Medical Center. Ann Intern Med. (2018) 168:801–8. doi: 10.7326/M18-0950, PMID: 29868815

[30] Cai S Fan J Zhu L Ye J Rao X Fan C. Bone mineral density and osteoporosis in relation to all-cause and cause-specific mortality in NHANES: a population-based cohort study. Bone. (2020) 141:115597. doi: 10.1016/j.bone.2020.115597, PMID: 32814125

[31] Shangguan X Xiong J Shi S Liao Y Chen L Deng J. Impact of the malnutrition on mortality in patients with osteoporosis: a cohort study from NHANES 2005–2010. Front Nutr. (2022) 9:868166. doi: 10.3389/fnut.2022.868166, PMID: 35634364 PMC9132007

[32] Gruzdeva O Borodkina D Uchasova E Dyleva Y Barbarash O. Localization of fat depots and cardiovascular risk. Lipids Health Dis. (2018) 17:218. doi: 10.1186/s12944-018-0856-8, PMID: 30219068 PMC6138918

[33] Koenen M Hill MA Cohen P Sowers JR. Obesity, adipose tissue and vascular dysfunction. Circ Res. (2021) 128:951–68. doi: 10.1161/CIRCRESAHA.121.318093, PMID: 33793327 PMC8026272

[34] Wang J Xing F Sheng N Xiang Z. Associations of the geriatric nutritional risk index with femur bone mineral density and osteoporosis in American postmenopausal women: data from the National Health and Nutrition Examination Survey. Front Nutr. (2022) 9:860693. doi: 10.3389/fnut.2022.860693, PMID: 35656160 PMC9152150

[35] Zhou D Liu X Huang Y Feng Y. A nonlinear association between body roundness index and all-cause mortality and cardiovascular mortality in general population. Public Health Nutr. (2022) 25:3008–15. doi: 10.1017/S1368980022001768, PMID: 35983642 PMC9991644

[36] Bolamperti S Villa I Rubinacci A. Bone remodeling: an operational process ensuring survival and bone mechanical competence. Bone Res. (2022) 10:48. doi: 10.1038/s41413-022-00219-8, PMID: 35851054 PMC9293977

[37] Wang L You X Zhang L Zhang C Zou W. Mechanical regulation of bone remodeling. Bone Res. (2022) 10:16. doi: 10.1038/s41413-022-00190-4, PMID: 35181672 PMC8857305

[38] Gandham A Mesinovic J Cervo MM Glavas C Jansons P Ng CA. Associations of body mass index, body fat percentage and sarcopenia components with bone health estimated by second-generation high-resolution peripheral quantitative computed tomography in older adults with obesity. Exp Gerontol. (2023) 179:112227. doi: 10.1016/j.exger.2023.112227, PMID: 37263367

[39] Zhao Z Yan K Guan Q Guo Q Zhao C. Mechanism and physical activities in bone-skeletal muscle crosstalk. Front Endocrinol. (2023) 14:1287972. doi: 10.3389/fendo.2023.1287972, PMID: 38239981 PMC10795164

[40] Piñar-Gutierrez A García-Fontana C García-Fontana B Muñoz-Torres M. Obesity and bone health: a complex relationship. Int J Mol Sci. (2022) 23:13662. doi: 10.3390/ijms23158303, PMID: 35955431 PMC9368241

[41] de Tejada-Romero MJG Saavedra-Santana P de la Rosa-Fernández F Suárez-Ramírez N Martín-Martínez A Del Rosario FM. Effect of obesity on fragility fractures, BMD and vitamin D levels in postmenopausal women. Influence of type 2 diabetes mellitus. Acta Diabetol. (2022) 59:1201–8. doi: 10.1007/s00592-022-01923-x, PMID: 35789433 PMC9329389

[42] Patil JD Fredericks S. The role of adipokines in osteoporosis management: a mini review. Front Endocrinol. (2024) 15:1336543. doi: 10.3389/fendo.2024.1336543, PMID: 38516409 PMC10956128

[43] Hetemäki N Mikkola TS Tikkanen MJ Wang F Hämäläinen E Turpeinen U. Adipose tissue estrogen production and metabolism in premenopausal women. J Steroid Biochem Mol Biol. (2021) 209:105849. doi: 10.1016/j.jsbmb.2021.105849, PMID: 33610799

[44] Liu Y Liu Y Huang Y Le S Jiang H Ruan B. The effect of overweight or obesity on osteoporosis: a systematic review and meta-analysis. Clin Nutr. (2023) 42:2457–67. doi: 10.1016/j.clnu.2023.10.013, PMID: 37925778

[45] Koskinas KC Van Craenenbroeck EM Antoniades C Blüher M Gorter TM Hanssen H. Obesity and cardiovascular disease: an ESC clinical consensus statement. Eur Heart J. (2024) 45:4063–98. doi: 10.1093/eurheartj/ehae508, PMID: 39210706

[46] Pin F Barreto R Kitase Y Mitra S Erne CE Novinger LJ. Growth of ovarian cancer xenografts causes loss of muscle and bone mass: a new model for the study of cancer cachexia. J Cachexia Sarcopenia Muscle. (2018) 9:685–700. doi: 10.1002/jcsm.12311, PMID: 30009406 PMC6104117

