# Supplementary material for: The L-shaped association between body roundness index and all-cause mortality in osteoporotic patients: a cohort study based on NHANES data
Source: Front Nutr. 2025 Jan 20;12:1538766. doi: 10.3389/fnut.2025.1538766 (PMC11788163; doi:10.3389/fnut.2025.1538766)
Supplement: Supplementary file 1 [file Table_1.docx]

**Table S1.Reference Population Bone Mineral Density T-Score**

| **Gender** | **Lumbar Mean** | **Lumbar Std Dev** | **Femoral Mean** | **Femoral Std Dev** |
| --- | --- | --- | --- | --- |
| **Male** | 1.049755447 | 0.116636241 | 0.940196903 | 0.129920763 |
| **Female** | 1.048036873 | 0.112443543 | 0.873960089 | 0.118126423 |

**Table S2.Association between BRI and all-cause mortality in osteoporotic population, excluding participants who died within the first two years of follow-up. (weighted)**

| **Characteristic** | **Model 1** |  |  | **Model 2** |  |  | **Model 3** |  |
| --- | --- | --- | --- | --- | --- | --- | --- | --- |
|  | **HR (95% CI)** | ***P* value** |  | **HR (95% CI)** | ***P* value** |  | **HR (95% CI)** | ***P* value** |
| All-cause mortality |  |  |  |  |  |  |  |  |
| BRI (continuous) | 0.99(0.94,1.06) | 0.9110 |  | 0.97(0.91,1.03) | 0.3447 |  | 0.91(0.84,0.98) | 0.0146 |
| BRI (category) |  |  |  |  |  |  |  |  |
| Low | Ref |  |  | Ref |  |  | Ref |  |
| High | 0.86(0.69,1.08) | 0.2050 |  | 0.71(0.57,0.88) | 0.0018 |  | 0.59(0.46,0.74) | <0.0001 |

Model 1, unadjusted; Model 2, adjusted for age group, gender, race; Model 3, adjusted for age group, gender, race, education level, family PIR, smoker, heavy drinker, calcium, 25-OHD, hypertension, dyslipidemia, cardiovascular disease, diabetes, liver disease, cancer, and kidney failure

**Table S3.Association Between BRI and All-Cause Mortality in Osteoporotic Adults. (Weighted, Non-Imputed Dataset)**

| **Characteristic** | **Model 1** |  |  | **Model 2** |  |  | **Model 3** |  |
| --- | --- | --- | --- | --- | --- | --- | --- | --- |
|  | **HR (95% CI)** | ***P* value** |  | **HR (95% CI)** | ***P* value** |  | **HR (95% CI)** | ***P* value** |
| All-cause mortality |  |  |  |  |  |  |  |  |
| BRI (continuous) | 0.99(0.92,1.08) | 0.9189 |  | 0.97(0.89,1.05) | 0.4619 |  | 0.90(0.82,0.99) | 0.0241 |
| BRI (category) |  |  |  |  |  |  |  |  |
| Low | Ref |  |  | Ref |  |  | Ref |  |
| High | 0.87(0.66,1.15) | 0.3281 |  | 0.73(0.57,0.93) | 0.0098 |  | 0.58(0.44,0.76) | <0.0001 |

Model 1, unadjusted; Model 2, adjusted for age group, gender, race; Model 3, adjusted for age group, gender, race, education level, family PIR, smoker, heavy drinker, calcium, 25-OHD, hypertension, dyslipidemia, cardiovascular disease, diabetes, liver disease, cancer, and kidney failure
